# Genotypic Characterization of Herpes Simplex Virus Type 1 Isolates in Immunocompromised Patients in Rio de Janeiro, Brazil

**DOI:** 10.1371/journal.pone.0136825

**Published:** 2015-09-25

**Authors:** Amanda Perse da Silva, Amanda de Oliveira Lopes, Yasmine Rangel Vieira, Adilson José de Almeida, Fernando Samuel Sion, Beatriz Grinsztejn, Sandra Wagner, Vanessa Salete de Paula

**Affiliations:** 1 Oswaldo Cruz Foundation, FIOCRUZ, Rio de Janeiro, Brazil; 2 Gaffrée & Guinle University Hospital, Federal University of the State of Rio de Janeiro (UNIRIO), Rio de Janeiro, Brazil; 3 Evandro Chagas National Institute of Infectology (INI), Rio de Janeiro, Brazil; Cincinnati Childrens Hospital Medical Center, UNITED STATES

## Abstract

Herpes simplex virus type 1 (HSV-1) is a prevalent human pathogen that causes a variety of diseases, including an increased risk of developing more severe disease in HIV-infected individuals. In Brazil, there is no information about the molecular epidemiology of HSV-1 infection, especially in HIV-infected individuals. The aim of this study was to perform the genotypic characterization of HSV-1 among HIV-infected patients. A total of 214 serum samples from HIV-positive patients without HSV infection symptoms were enrolled in one of two reference hospitals for HIV infection managing in Rio de Janeiro. The gG and gI genes were analyzed by restriction fragment length polymorphism (RFLP) and full nucleotide sequencing of the US8 (1601 bp), UL44 (1996 bp), and UL23 (1244 bp) regions was performed. A total of 38.3% (82/214) and 32.7% (70/214) of the serum samples tested positive for gG and gI genes, respectively. RFLP analysis classified the HSV-1 as belonging to genotype A. Phylogenetic analysis of the Brazilian samples for the US8, UL44, and UL23 regions demonstrated that the nucleotide identity between Brazilian samples was higher than 97% for all genes. No acyclovir mutation was detected in the patients. The shedding of HSV in the serum samples from HIV-positive patients who were asymptomatic for HSV infection was detected in this work. This is the first report of molecular characterization of HSV-1 in Brazilian samples since there is no previous data available in the literature concerning the genotypic classification and stable distribution of Brazilian strains of HSV-1 in Rio de Janeiro, Brazil.

## Introduction

Herpes simplex infection is caused by either Herpes simplex virus type 1 (HSV-1) or Herpes simplex virus type 2 (HSV-2), with tropism for mucosa surfaces where it causes skin vesicles or mucosal ulcers or it can simply be excreted in the absence of symptoms [1]. Herpes simplex virus (HSV) infections contribute substantially to hospitalization, morbidity, and mortality in HIV-infected patients [2].

HSV-1 is one of the most common viruses in humans, and infection caused by this agent occurs often during childhood and adolescence [3]. Some studies have demonstrated an increasing frequency of HSV-1 as the agent of genital herpes in developed countries [4, 5]. HSV-2 is more prevalent in sexually active adults and adolescents [6, 7]. In most geographic regions, the prevalence of HSV-1 infection is greater than HSV-2 infection [8].

HSV-1 infections may occur in a primary or a recurrent form and may lead to substantial physical morbidity [3]. In primary infection, the replication of the virus at the portal of entry, usually oral or genital mucosa, results in infection of sensory nerve endings [1]. The ability of the virus to replicate in mucosa and to be transported to the dorsal root ganglia is associated with the pathogenesis of the infection. Primary infection can spread beyond the dorsal root glanglia, thereby becoming systemic. HSV-1 is able to establish a lifelong latent infection of sensory ganglia with intermittent reactivation and neuronal spread of the virus to innervating tissues [9]. HSV-1 can be reactivated from latency [10] and, during reactivation, the virus is detected at mucocutaneous sites, and appears as skin vesicles or mucosal ulcers, or it can be excreted without any symptoms [1].

Information about the frequency and clinical correlates of HSV viremia is limited. No HSV viremia has been detected in patients with recurrent herpes labialis but otherwise in healthy individuals. Thus, the detection of HSV viremia is possible, but seems to be limited to primary infections since it has not been detected in recurrent infections [11]. In a previous study to evaluate HSV viremia during primary genital infection, 24% of the patients had HSV DNA detected when assayed by polymerase chain reaction (PCR) in their peripheral blood [12].

HSV-1 reactivation is more common in immunocompromised individuals and may result in viral shedding in saliva [13]. Patients with immunodeficiencies or treatment-related immunosuppression are at increased risk of developing severe generalized or prolonged HSV excretion [14]. PCR-based assays can rapidly and accurately detect and quantify HSV DNA from clinical specimens of lesion, saliva, serum, and plasma [15–17].

The genome of HSV consists of two components that are formed from unique sequences (U) covalently bound to either L (long) or S (Short) sequences, and are identified in relation to the size of the genomic fragment, using the abbreviations UL or US [18, 19]. HSV-1 is a double-stranded DNA virus with a 152 kb genome that encodes at least 74 proteins [14]. Molecular recombination generates new combinations of genetic material. These mechanisms are poorly understood but are associated with DNA replication [20] and different cell factors [21, 22].

Acyclovir (ACV) is one of the antiviral drug indicated for the treatment of herpes infections. ACV acts through the inhibition of viral DNA polymerase (pol) by acting as a competitive inhibitor of viral DNA. Initial phosphorylation of ACV by viral thymidine kinase (TK) in infected cells provides this competitive inhibition [23]. Resistance to ACV in the majority of cases (95%) is due to a mutation in TK or by alteration in substrate specificity [24, 25].

The aim of this study was to detect and molecularly characterize the circulating strains of HSV-1 in HIV-positive patients. Many studies on HSV-1 phylogeny have analyzed the viral strains in different geographic regions such as Europe, North America, East Asia, and Eastern Africa [26, 27]. These studies have described three genotypes for HSV-1 (A, B, and C) when analyzed through the glycoprotein E (gE), C (gC), and I (gI) [10, 28]. In Brazil, until now, there are no data available concerning the genotypic characterization of circulating HSV-1 isolates.

## Materials and Methods

### Ethics Statement

This study was approved by the Human Research Ethics Committee of Evandro Chagas National Institute of Infectology (protocol number: 0039.0.009.000–10) and Gaffrée & Guinle University Hospital (protocol number: 11350212.3.0000.5258). All the subjects participating in the study signed a consent form after being provided all the necessary and sufficient information to make an informed decision.

### Study Population

In this study, 214 serum samples were tested for HSV-1. Between January 1988 and December 2012, a total of 173 serum samples of HIV-infected patients followed up at Gaffrée & Guinle University Hospital of the Federal University of the State of Rio de Janeiro were collected. In the course of 2012, 41 serum samples of HIV-infected patients followed up at the Evandro Chagas National Institute of Infectology of Oswaldo Cruz Foundation were also collected. Both Institutions are Public Health Care Units localized in Rio de Janeiro, Brazil. Inclusion criteria were as follows: 18 years old or older, male or female, any race, HIV infection confirmed by enzyme-linked immunosorbent assay and Western blot. All patients included were asymptomatic for HSV-1.

At Gaffrée & Guinle University Hospital 61.8% (107/173) of patients were males, with a median age of 45 years old (ranging from 18 to 72 years old). At Evandro Chagas National Institute of Infectology, 68.29% (28/41) of patients were males, with a median age of 40 years old (ranging from 21 to 60 years old).

### HSV-1 detection and quantification

Viral DNA was isolated from serum samples using the QIAmp DNA Mini kit (QIAGENValencia, CA, USA) according to the manufacturer's instructions. Extracted DNA was analyzed by qualitative PCR specific for gG (269 bp) and gI (410 bp) for partial genome amplification. The following reagents were used in the PCR assays: 5x Phusion Buffer, 1X Phusion DNA Polymerase (Phusion High Fidelity DNA Polymerase, New England BioLabs, MA, USA), primers 10 μM (forward/ reverse), dNTPs 20 μM, and 5 μL of DNA. Single-positive samples were tested by restriction fragment length (RFLP), according to published protocols [28, 29].

In order to quantify the viral load, DNA samples were evaluated in duplicate by Real Time PCR using Real StarHSV PCR Kit 1.0 Multiplex (Altona Diagnostic, Hamburg, Germany), according to manufacturer’s instructions. This kit contains all the components (buffer, enzymes, primers, and probes) to allow simultaneous detection and quantification of HSV-1 and HSV-2.

### Partial genome amplification and direct nucleotide sequencing

The US8 (1601 bp), UL44 (1996 bp), and UL23 (1244 bp) regions were subjected to partial genome amplification under the conditions established in this study: 20 μL reaction volume consisted of 2 μL DNA, HotStar Taq Plus Master Mix 1X (QIAGENHilden, Germany), primers 0.3 μM (forward/ reverse), and H_2_O RNAse free. Cycling conditions were as follows: initial denaturation at 95°C for 5 min, followed by 45 cycles of denaturation at 95°C for 30 s, annealing at 55°C for 45 s and extension at 72°C for 90 s, and a final extension at 72°C for 10 min. Amplicons were analyzed by electrophoresis on 2.5% agarose gel, stained with ethidium bromide (0.2 μg/mL) and observed under UV light.

PCR products with expected size were purified using reagents and protocols of the QIAquick Gel Extraction kit (QIAGEN, Hilden, Germany) and sequenced using reagents and protocols of the ABI Kit Big DyeTerminator version 1.1 cycle sequencing kit (Applied Biosystems, Foster City, CA,USA) at the sequencing platform of LGC Genomics. Samples were sequenced using primers previously used in PCR reactions (Table 1).

**Table 1:** Primers used for amplification and sequencing of the UL23, UL44, and US8 genes of HSV-1.

Gene and primer orientation*	Sequence (5´→3´)	Fragment length (bp)
**UL-23**		
**UL-23 1F**	CGTTATTTACCCTGTTTCG	290
**UL-23 272R**	ATTGTCTCCTTCCGTGTT	290
**UL-23 253F**	CCGAAACAGGGTAAATAACG	468
**UL-23 720 R**	GTTCTGGCTCCTCATATCG	468
**UL23 702F**	CGATATGAGGAGCCAGAAC	486
**UL23 1187R**	ATCTTGGTGGCGTGAAAC	486
**UL-44**		
**UL44 1F**	AGGCATTAGTCCCGAAGA	573
**UL44 331R**	TGGTGTTGTTCTTGGGTTT	573
**UL44 311F**	CCAAACCCAAGAACAACAC	392
**UL44 702R**	CACCTCGCCGATAATCAG	392
**UL44 643F**	CCTCCGTTGTATTCTGTCA	404
**UL44 1104R**	ATCTGGCAGGTGAAGGTC	404
**UL44 1090F**	CTTCACCTGCCAGATGAC	627
**UL44 1551R**	GGATCGACCAAGGATGAC	627
**US-8**		
**US8 21F**	TTCTTCTCGGTGTTTGTGTTG	657
**US8 739R**	TCTCCATACGCACGGTCAC	657
**US8 724F**	ACCGTGCGTATGGAGACT	440
**US8 1163R**	GCGTGAATATGGTCGTTGA	440
**US8 1144F**	GTCAACGACCATATTCACG	564
**US8 1707R**	CCAGAAGACGGACGAATC	564

*F, forward; R, reverse; bp, base pairs.

### Phylogenetic analysis

HSV-1 sequences were manually checked in the BioEdit. Bayesian phylogenetic trees were constructed with partial nucleotide sequences from positive samples for US8 (1601 bp), UL44 (1996 bp), and UL23 (1244 bp), as well as sequences from GenBank. Consensus trees were visualized using the FigTree program. A nucleotide identity matrix was also constructed using the Brazilian strains and nucleotide sequences available in the literature.

For the US8 region, multiple nucleotide sequence alignment was analyzed by using the Markov Chain Monte Carlo method implemented in the program MrBayes version 3.1.2 under the HKY + γ+ I nucleotide substitution model, selected by using the jModeltest program (ngen = 8,000,000). For the UL44 region, multiple nucleotide sequence alignment was analyzed by using the Markov Chain Monte Carlo method implemented in the program MrBayes version 3.1.2 under the GTR + γ nucleotide substitution model, selected by using the jModeltest program (ngen = 10,000,000). For the UL23 region, multiple nucleotide sequence alignment was analyzed by using the Markov Chain Monte Carlo method implemented in the program MrBayes version 3.1.2 under the GTR + γ+ I nucleotide substitution model, selected by using the jModeltest program (ngen = 12,000,000). Nucleotide sequence accession numbers are indicated below.

### Nucleotide Sequence Accession Numbers

#### US8

BR- I15- KM279031, BR8-KM279032, BR61- KM279033, BR60-KM279034,BR54-KM279035, BR52- KM279036, BR19-KM279037, BR18- KM279038, BR16- KM279039, BR15- KM279040, BR12-KM279041, BR 9-KM279042, BR6- KM279043, BR3-KM279044, BR67-KM279045, BR5-KM279046, BR I18- KM279047, BR23- KM279048, BR-10- KM279049, BR 4- KM279050

#### UL23

BR I15- KM279052, BR68- KM279053, BR61- KM279054, BR60- KM279055, BR54 KM279056, BR 52- KM279057, BR19- KM279058, BR18- KM279059, BR16- KM279060, BR15-KM279061, BR12- KM279062, BR9-KM279063, BR6-KM279064, BR3- KM279065, BR67-KM279066, BR5-KM279067, BR I18- KM279068, BR 23-KM279069, BR10-KM279070, BR4-KM279071,KOS_UL23- KM279051.

#### UL44

BR5- KM279072, BR I18- KM279074, BR I15- KM279075, BR68- KM279076, BR67- KM279077, BR61- KM279078, BR60- KM279079, BR54-KM279080,BR52-KM279081, BR19-KM279082, BR18-KM279083, BR16- KM279084, BR15- KM279085, BR10- KM279086, BR 9-KM279087, BR6- KM279088, BR4- KM279089, BR3-KM279090, BR23-KM279091, BR12-KM279092

## Results

### HSV-1 detection and restriction fragment length (RFLP)

HSV-1 DNA was detected in serum samples from HIV-infected patients. In the study population 38.3% (82/214) and 32.71% (70/214) of the samples showed a positive result for PCR specific for gG (269 bp) and gI (410 bp) genes, respectively. Also, 22.8% (49/214) of the patients showed positive result for both the gG and the gI genes.

Positives samples for HSV-1 detection were subjected to genotypic classification based on the RFLP technique, according to published protocols [28, 29]. For the gG gene, the amplicon was not cleaved for isolates belonging to the genotype A, but two fragments were generated (97 and 172 bp) for isolates belonging to the genotype B, and three fragments were generated (57, 97, and 115 bp) for isolates belonging to the genotype C. For gI gene, the amplicon was not cleaved for isolates belonging to the genotype A, however, two fragments were generated (55 and 355 bp) for genotype B isolates, and three fragments were generated (55, 133, and 222 bp) for genotype C isolates. The RFLP assay demonstrated that 71.95% (59/82) and 61.42% (43/70) of the tested samples confirmed the positive results for gG and gI genes, respectively. All Brazilian strains analyzed in the study were assigned to genotype A for the two genes (gG and gI).

### Real Time PCR

Real Time PCR included 214 serum samples of the patients. A linear relationship was obtained between the cycle threshold (Ct) values and the log_10_ concentration of the HSV-1/2 DNA. The regression analysis yielded a correlation coefficient of 0.99. In this analysis, 29% (62/214) of the serum samples were positive for HSV-1. Viral loads ranged from 2.75 x10^2^ to 8.78 x 10^3^ copies/mL. None of the analyzed samples tested positive for HSV-2.

### Phylogenetic analysis

Twenty serum samples were selected for partial genome amplification of the US8, UL44, and UL23 regions and direct sequencing. Results of samples from HIV-positive patients are shown in Table 2. All HIV-positive patients had been under antiretroviral therapy (ART) for at least one year.

**Table 2:** Characteristics and molecular information of HIV/HSV-positive samples.

Samples	Gender	Year of collection	RFLP gG	RFLP gI	Real Time (copies/mL)
BR3	Male	2004	Genotype A	Genotype A	2.05 x10^3^
BR4	Female	2004	Genotype A	Genotype A	1.98 x 10^3^
BR5	Male	2004	Genotype A	Genotype A	2.72 x10^3^
BR6	Female	2004	Genotype A	Genotype A	1.01 x 10^3^
BR9	Female	2004	Genotype A	Genotype A	4.26 x10^3^
BR10	Female	2004	Genotype A	Genotype A	8.78x10^3^
BR12	Male	2003	Genotype A	Genotype A	1.43x10^3^
BR15	Male	2003	-	Genotype A	8.65x10^3^
BR16	Male	2003	Genotype A	Genotype A	1.57x10^3^
BR18	Male	2003	Genotype A	Genotype A	1.06x10^3^
BR19	Male	1988	Genotype A	-	2.52x10^3^
BR23	Female	2000	Genotype A	Genotype A	2.89x10^3^
BR52	Female	1997	Genotype A	Genotype A	1.12x10^3^
BR54	Male	1997	Genotype A	Genotype A	1.17x10^3^
BR60	Male	2004	-	Genotype A	3.56x10^3^
BR61	Male	1988	-	Genotype A	5.29x10^3^
BR67	Female	2004	Genotype A	Genotype A	4.77x10^3^
BR68	Male	2004	Genotype A	Genotype A	4.04x10^3^
BR I15	Male	2012	-	Genotype A	2.94x10^2^
BR I18	Male	2012	-	-	2.75x10^2^

**Fig 1:**
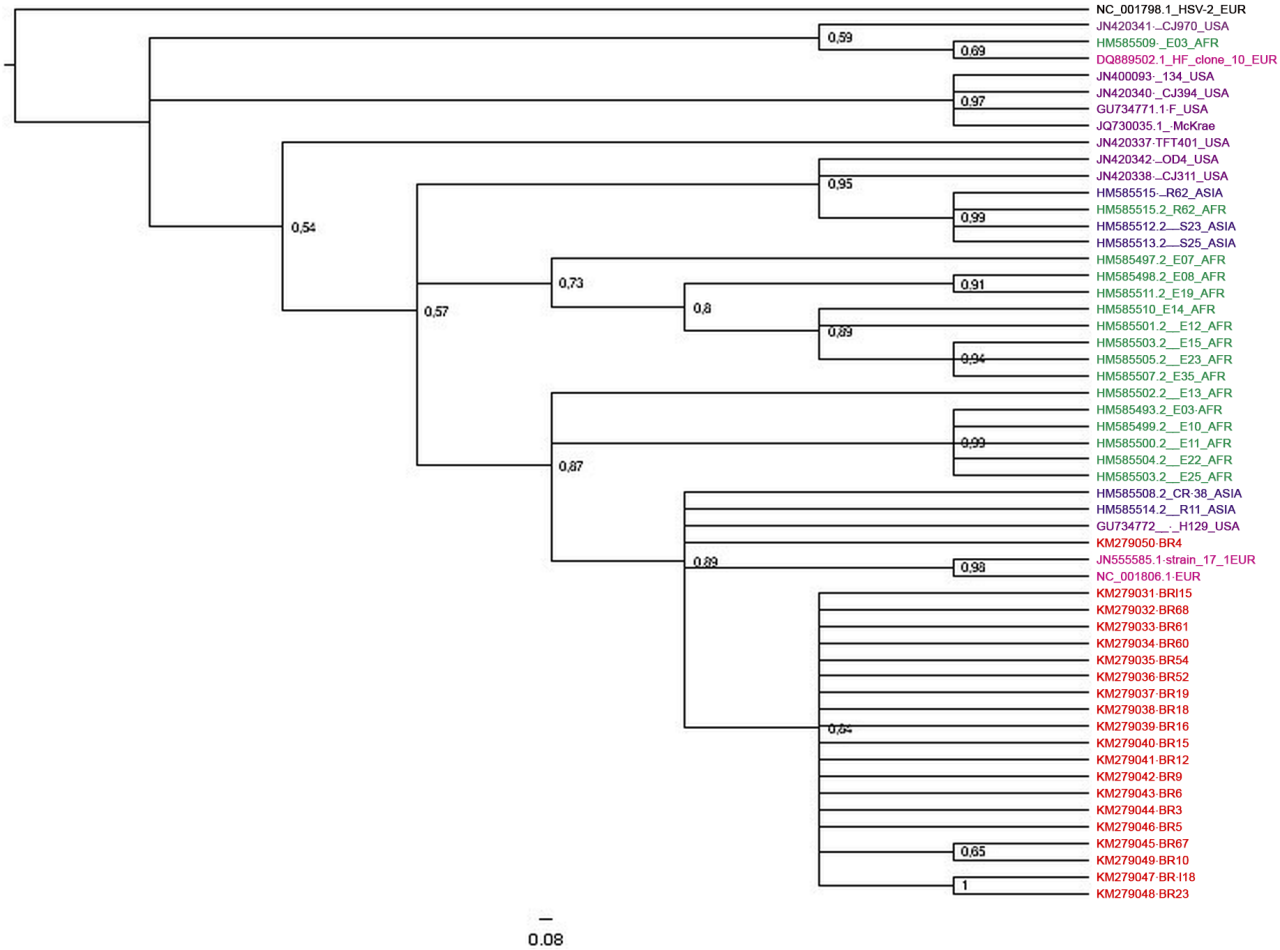
The Bayesian phylogenetic tree constructed by using nucleotide sequence of US8 coding region (1601 bp). The GenBank accession number is shown for each sequence used. Posterior probabilities are shown at the branch label. Brazilian sequences are noted in red. Color code: Africa—Green, Asia—Blue, Europe—Pink, United States—purple, Brazil—red.

Phylogenetic reconstruction using partial nucleotide sequences of US8 (1601 nt) showed a close relationship of the HSV-1 Brazilian strains from HIV-infected patients with the HSV-1 nucleotide sequences from Europe, North American, African and Asian strains (Fig 1). Thirty-four previously sequenced HSV-1 strains were included in this analysis based on geographic origins [26, 27].

As demonstrated for US8, the Brazilian sequences were aggregated in the same clade. The genetic distances calculated using the nucleotide sequences of US8 (1601 nt) showed a phylogenetic distance between the virus detected in the Brazilian serum samples and the Genbank database listed by NCBI Blast (H129 USA, Strain 17 EUR, NC 001806 EUR) (Fig 1). The nucleotide identity values of Brazilian strains when compared to those strains ranged from 99.5% to 99.9%.

**Fig 2:**
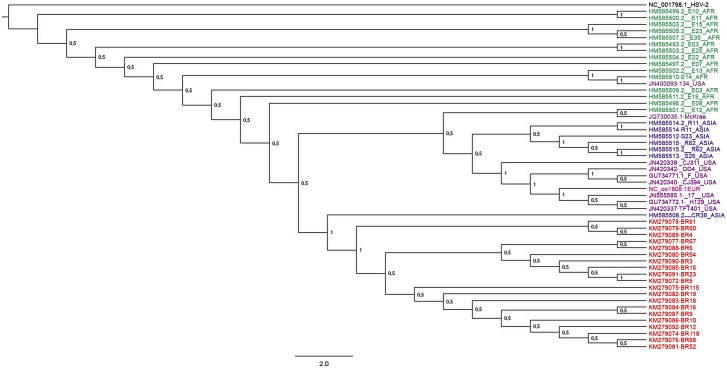
The Bayesian phylogenetic tree constructed by using nucleotide sequence of UL44 (1996 bp). The GenBank accession number is shown for each sequence used. Posterior probabilities are shown at the branch label. Brazilian sequences are noted in red. Color code: Africa—Green, Asia—Blue, Europe—Pink, United States—purple, Brazil—red.

Phylogenetic reconstruction using partial nucleotide sequences of UL44 (1996 nt) is shown in Fig 2. In this analysis, all Brazilian samples aggregated in the same clade. The nucleotide identity values between Brazilian samples for genes US8 and UL44 were higher than 98%. Genetic distances calculated with nucleotide sequences of UL44 (1996 nt) showed a significant phylogenetic distance between the virus detected in Brazilian serum samples and CR38 sample of Asian origin. The nucleotide identity values between Brazilian strains and this strain ranged from 99.4% to 99.9%.

Phylogenetic reconstruction using partial nucleotide sequences of UL23 (1244 nt). The UL23 gene codifies the viral thymidine kinase (TK) and mutations in TK result in resistance to acyclovir (AVC). The mutation in HSV TK result in 95% of the cases of ACV-resistance [30]. These mutations consisted of nucleotide insertions\deletions leading to the shift of the translational reading frame of the UL23 gene, and half of them were in amino acid substitutions [31, 32]. Based on the nucleotide identity analysis, the values found among the Brazilian samples were 98.4–100% and the nucleotide identity values from different samples described in the Genbank were 97.5%-99.9% (S1 Fig).

## Discussion

Infections caused by HSV-1 and HSV-2 are among the most prevalent viral infections in the world [33]. The HIV/AIDS epidemic is present worldwide and approximately 33 million people are currently living with HIV/AIDS. In Brazil, according to the report of the Epidemiologic Newsletter in 2013 there were 656,701 reported cases of AIDS [34]. Opportunistic infections due to immune deficiency have been recognized as the most common complications of HIV infection and are responsible for hospitalizations and substantial morbidity in infected patients.

The treatment with anti-HSV drugs in HIV-1-positive patients reduces HIV plasma levels and the occurrence of HSV genital ulcers. However, clinical trials of suppressive therapy for HSV-2 (acyclovir) in HSV-2 seropositive individuals have failed to reduce the risk of transmission of HIV-1 to their partners[35, 36].

In our study, HSV DNA was detected in serum from asymptomatic HIV-positive patients, with a viral load ranging from 2.75 x 10^2^ to 8.78 x 10^3^ copies/mL, showing the shedding of HSV in serum samples of these patients. Detection of HSV DNA using PCR from genital lesions frequently demonstrates HSV in the genital tract of both asymptomatic and symptomatic individuals[37]. In a study with hospitalized patients, HSV DNA was detected by PCR in the peripheral blood of patients with unexplained sepsis syndrome, evidence of hepatitis, and/or CNS infection with fever. Approximately 1/4 of the patients who had HSV detected in the peripheral blood were immunocompetent, and only a minority of patients had mucocutaneous lesions [38]. Despite data provided by PCR, little is known about the frequency and clinical correlates of HSV viremia. Viral dissemination to the bloodstream and visceral disease can be seen in neonates [39] and immunocompromised patients, including those with hematologic malignancies [40] and transplant recipients [41, 42]. HSV viremia is possible, but seems to be limited to primary infections as it has not been detected in a recurrent infection [11]. Unfortunately, in our study it was not possible to know whether patients presented the primary HSV infection. Therefore, more studies are necessary to determine the true prevalence and clinical significance of qualitative and quantitative detection of HSV DNA in serum and/or peripheral blood of the HIV-positive patients.

HSV DNA detection was confirmed by RFLP analysis of the gG and gI genes and through a complete sequencing of the US8, UL44, and UL23 genes. The study on HSV-1 sequences using RFLP can be used for screening and genotyping. RFLP analysis demonstrated the stable presence of genotype A in the Brazilian samples. Previous studies revealed that HSV-1 strains can vary according to geographic region [27] and demonstrated the stable presence of genotype A in the majority of strains [43].

Recent studies by Kolb in 2013 and Spzara in 2014 [26, 27] demonstrated the presence of geographical clustering and they divided the samples into three continental groups (Asia, Africa, North America and Europe) after whole genome analysis. Although these studies have shown that multiple genomes can be sequenced at the same time using the Illumina platform [26, 27], the sequences of the HSV-positive samples were isolated from cell culture. In our study, HSV-1 DNA was directly isolated from serum samples with low viral load. Concerning the US8 gene, previous phylogenetic analysis revealed three main genetic groups, the A, B, and C genogroups, demonstrating the presence of tree subclades and confirmed their European origin [26, 44]. The Brazilian samples were compared to samples described in Europe, North America, Africa and Asia and were classified into the group A. In the analysis of the US8 region, the Brazilian samples were grouped, with exception of the strain KM279050-BR4 that was more related to samples described in Europe, United States, and Asia. Phylogenetic studies on the short unique region of the genome demonstrated a three clade pattern for HSV-1 in US8 gene, as well as in US1, US4, and US7 [29, 45, 46], in which the classification was supported by high bootstrap values [29].

The US and UL regions have a large number of recombination events attributed to the distance between these two regions. In our study, phylogenetic analysis of UL44 showed that the Brazilian samples were aggregated in the same clade with nucleotide variations at position 1114 T>C, and changes in the position of the amino acids were also observed. The analysis of the US8 and UL44 genes separated the Brazilian samples into clades with high posterior probabilities (pp).

Information on mutations in the TK and DNA pol genes of HSV-1 are relevant for antiviral treatment outcomes[3], especially in immunocompromised patients. HSV isolates resistant to ACV have no clinical relevance in immunocompetent persons, however, the prevalence of HSV infections with reduced susceptibility to ACV varies between 3.5% and 7.1% in immunocompromised patients[47]. The best method to verify the unique role of mutations that contribute to HSV resistance is genotype analysis of the viral isolates [3] and the sequencing of UL23 gene, that is highly conserved [48]. In our study, the nucleotide identity values between the Brazilian strains were higher than 98% and mutations in the TK gene that might confer resistance to ACV were not found in the sequenced HSV strains.

Both techniques used in the present study, RFLP and nucleotide sequencing, demonstrated the presence of a unique HSV-1 genotype distribution that has been stable within Brazil for 24 years (1988 to 2012); phylogenetic analysis using US8, UL44, and UL23 demonstrated that the Brazilian samples (South American) are aggregated into a single clade with only Brazilian strains. The genotypic characterization of HSV-1 isolates from patients enrolled in our study reflects the HIV-positive patients of the Rio de Janeiro population, thus not reflecting as a whole the diversity of patients living in other regions of the country. The samples from Rio de Janeiro patients were related to each other with high posterior probabilities in all genes analyzed. Data on the prevalence of HSV-1 in Germany demonstrated a stable distribution of the A and B genotypes for a period of 10 years [10]. A study based on the quantitative assessment of genomic polymorphisms in strains of HSV-1 in six countries, three Asian and three non-Asian in origin, concluded that the evolutionary pattern was similar between these ethnic groups, and that the variability found in Asian samples was lower when compared to non-Asian samples [49]. HSV-1 phylogenetic analysis studies have suggested a substitution rate of approximately 1.38 x 10^−7^ subs/site/year [26]. By comparing Brazilian samples with other samples described in the Genbank, we found nucleotide identity values between 96% and 100% for the US8, UL44, and UL23 genes.

In conclusion, our study showed the circulation of HSV-1 in HIV-positive patients without HSV infection symptoms, which confirmed HSV shedding in immunocompromised patients and demonstrated that Brazilian isolates are grouped in the same clade with a low nucleotide variation in the genes US8, UL44, and UL23.

## Supporting Information

S1 Fig The Bayesian phylogenetic tree constructed by using nucleotide sequence of UL23 region. The GenBank accession number is shown for each sequence used. Posterior probabilities are shown at the branch label. Brazilian sequences are noted in red.
(TIF)
