# Supplementary figures and images for: Genotypic Characterization of Herpes Simplex Virus Type 1 Isolates in Immunocompromised Patients in Rio de Janeiro, Brazil
Source: PLoS One. 2015 Sep 25;10(9):e0136825. doi: 10.1371/journal.pone.0136825 (PMC4583264; doi:10.1371/journal.pone.0136825)

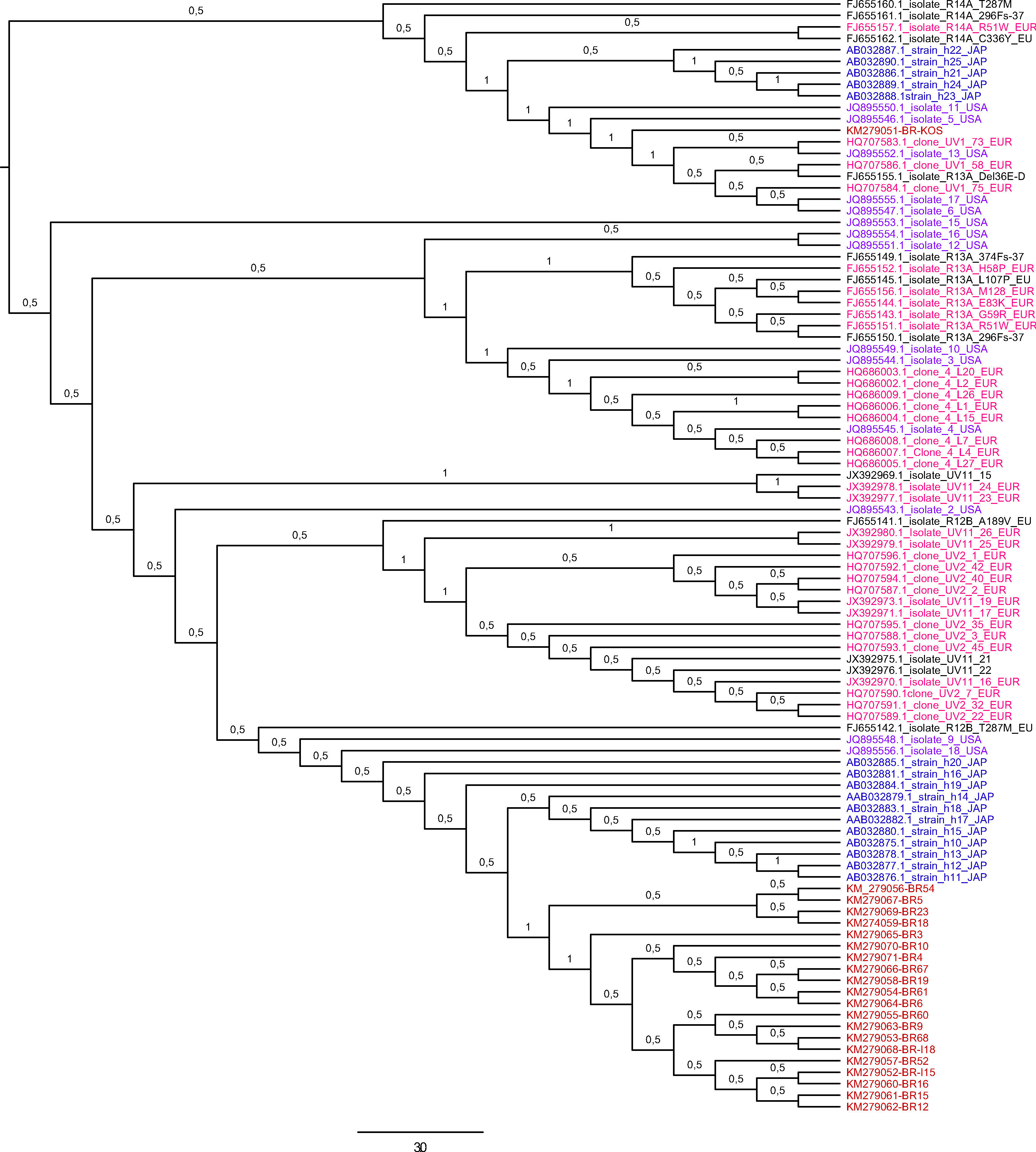

Supplement: S1 Fig — The GenBank accession number is shown for each sequence used. Posterior probabilities are shown at the branch label. Brazilian sequences are noted in red. (TIF) [file pone.0136825.s001.tif]
